# Exosomes in Genitourinary Cancers: Emerging Mediators of Drug Resistance and Promising Biomarkers

**DOI:** 10.7150/ijbs.78321

**Published:** 2023-01-01

**Authors:** Zeyi Lu, yuanlei Chen, Wenqin Luo, Lifeng Ding, Huan Wang, Yang Li, Bo wen Yang, Liangliang Ren, Qiming Zheng, Haiyun Xie, Ruyue Wang, Chenhao Yu, Yudong Lin, Zhenwei Zhou, Liqun Xia, Gonghui Li

**Affiliations:** Department of Urology, Sir Run Run Shaw Hospital, Zhejiang University School of Medicine, Hangzhou, China.

**Keywords:** exosome, genitourinary cancers, drug resistance

## Abstract

Drug resistance presents a major obstacle in the treatment of genitourinary cancers. Exosomes as the medium of intercellular communication serve important biological functions and play essential roles in pathological processes, including drug response. Through the transfer of bioactive cargoes, exosomes can modulate drug resistance via multiple mechanisms. This review attempts to elucidate the mechanisms of exosomal cargoes with reference to tumor drug resistance, their role in genitourinary cancers, and their potential clinical applications as candidate biomarkers in liquid biopsy.

## 1. Introduction

Genitourinary cancers are considered to be a group of specific malignancies found in the urinary system and the male reproductive system, with the most common subtypes of renal cell carcinoma (RCC), urothelial carcinoma of the bladder (UC) and prostate carcinoma (Pca), predominantly affecting male individuals. It is estimated that there will be over 432,680 new cases and 66,490 deaths caused by genitourinary cancer in the United States in 2022. Genitourinary cancer leads to 39% of all incident cases caused by cancer in men in the United States, among which prostate cancer alone accounts for 27% of diagnoses^1^. Although improvements in earlier diagnosis and treatments contributed to the declined age‐standardized cancer mortality for Pca (-11.9%), UC (-1.5%) and kidney cancer (-0.9%) from 1971 to 2019, genitourinary cancer still resulted in 15% of estimated cancer deaths in males^2^. In clinical practice, surgical treatment is recommended for early or localized genitourinary malignancies, including partial nephrectomy for RCC 3, transurethral resection of bladder tumors 4, and transurethral resection of the prostate (TURP) for Pca 5. However, surgical treatments are no longer suitable for advanced stage or metastatic stage cancers, considering their weak effect on patients' prognosis. Instead, systemic approaches, including chemotherapeutic, hormonal, targeted, and immune therapy, are considered to be first-line treatments that could effectively improve survival rates. However, the development of tumor drug resistance has gradually become a major burden affecting patient prognosis. Predicting the efficacy against tumors before dosing and overcoming tumor resistance after dosing have emerged as the major research directions in current clinical trials. Therefore, clarifying the mechanisms underlying drug resistance and seeking the corresponding biomarkers to predict the therapeutic effect are urgent tasks to improve the treatment options for genitourinary cancers.

Over the years, exosomes have emerged as a primary research subject according to their rapidly evolving role spanning a variety of fields. Exosomes are one of the subsets of extracellular vehicles (EVs) ranging between 30-150 nm in diameter, which are ubiquitously distributed in organisms. Initially, researchers regarded exosomes as part of a waste-cleaning system, responsible for packaging and secreting metabolic waste outside. However, recent studies have proved that exosomes are an important part of intercellular communication, mediating the development of various physiological processes and performing extremely important biological functions 6. A growing number of studies have reported that exosome-derived proteins or non-coding RNAs play important roles in tumor drug resistance 7-9.

The mechanisms underlying exosome-mediated drug resistance can be specific according to the various bioactive cargoes. In this review, we summarize the recent advances in the association of exosomal cargoes with drug resistance in genitourinary cancers (Table 1). In addition, we discuss the potential role of exosomes as cancer biomarkers to predict therapeutic responses and tumor progression.

## 2. Biogenesis and characteristics of exosomes

**Figure 1:**
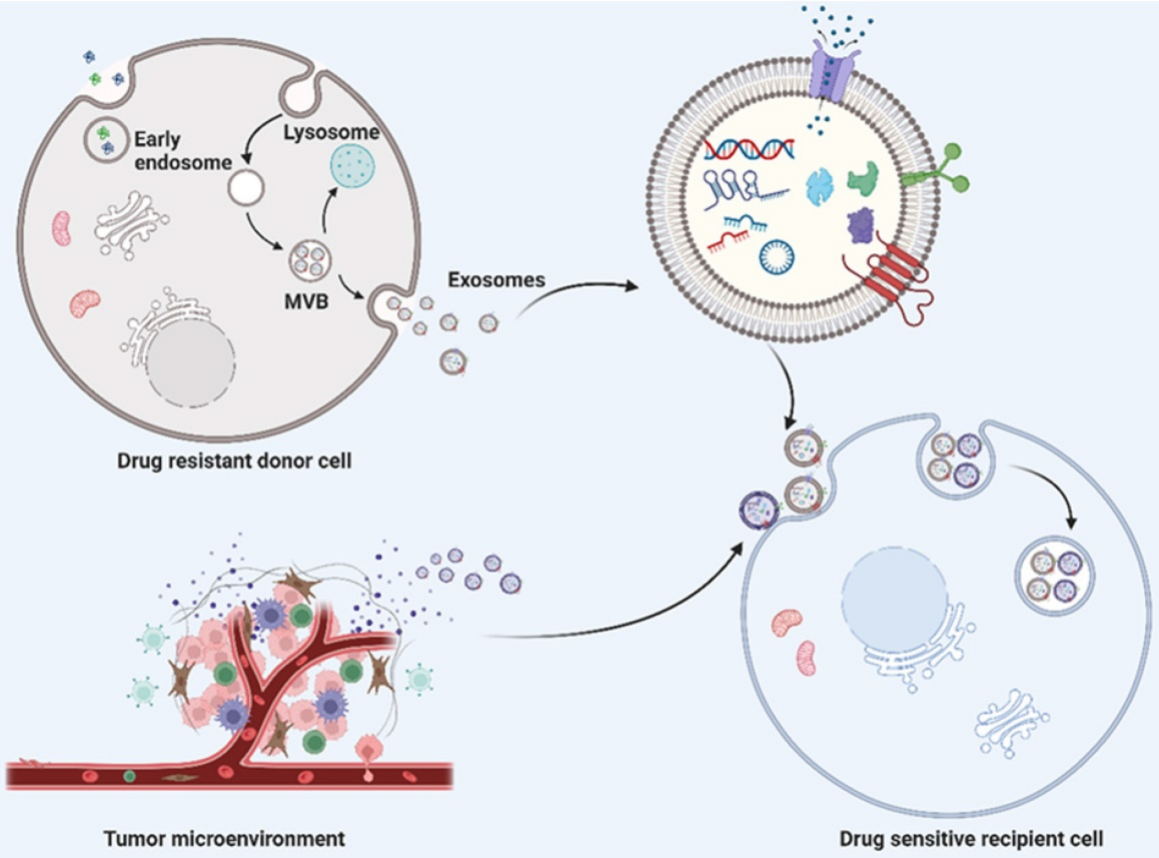
** Schematic description of exosome biogenesis and transportation of exosome cargoes from drug resistant donor cells and their microenvironment to drug sensitive recipient cells.** The double invagination of plasma membrane generates intracellular multivesicular bodies (MVBs) and intraluminal vesicles (ILVS, future exosomes) inside the MVBs. The mature MVBs can either fused with plasma membrane to release the inner ILVS as exosomes, or fused with lysosomes or autophagosomes for degradation. Exosomes derived from drug resistant donor cells and tumor microenvironment transport their cargoes to drug sensitive recipient cells, thereby inducing drug resistance. Exosomal cargoes, including nucleic acid (such as DNA, mRNA and noncoding RNAs) and proteins indeed play a significant role in transferring drug resistance phenotype to drug‐sensitive cells. Created with BioRender.com.

Exosomes are single-membrane EVs originating from endosomal membranes, which have a nearly ubiquitous distribution in the microenvironment 10. The formation of exosomes requires the double invagination of the plasma membrane, which produces intracellular multivesicular bodies (MVBs) and intraluminal vesicles (ILVS, future exosomes) inside the MVBs. Mature MVBs can be either fused with the plasma membrane to release the inner ILVS as exosomes or fused with lysosomes or autophagosomes to be degraded (Fig. 1). The formation of exosomes involves the endosomal sorting complex required for transport (ESCRT), which mediates the sorting and loading of exosome contents. The cargo of exosomes can be broadly divided into proteins (membrane proteins, cytosolic and nuclear proteins, extracellular matrix proteins), metabolites and nuclear acids (mRNA, noncoding RNA species, and DNA) 11-13. The exosome content can be altered in accordance with the biological function mediated by the exosome. With regard to the characterization of exosomes, the International Society of Extracellular Vesicles (ISEV) recommends at least three protein markers for the measurement of exosome origin, containing one transmembrane protein (e.g., tetraspanins and integrins), one cytosolic protein (e.g., programmed cell death 6 interacting protein (also known as ALIX) and tumor susceptibility 101 (TSG101), and one negative protein marker 6, 14, 15.

## 3. Exosomal cargoes mediating tumor drug resistance

Exosomes represent a principal medium of cellular communication between cells and their surrounding microenvironment. Depending on the exosome-mediated transmission of specific cargoes, corresponding physiological or pathological function changes will be induced in the recipient cells. Studies have revealed the emerging role of exosomes contributing to the acquirement of tumor drug resistance 9, 16. In response to exosomes derived from the tumor microenvironment and drug-resistant tumor cells, a series of pathological changes will be then promoted in the reicipent malignant cells, such as activating alternative anti-apoptotic signaling pathways, inducing epithelial-mesenchymal transition (EMT), affecting intracellular drug metabolism, etc., which eventually result in drug-resistance 17, 18. Accordingly, the diverse roles of exosomes in tumor drug resistance can be roughly categorized depending on the cargoes loaded (Fig. 1).

### 3.1. Exosomal DNA cargoes and tumor drug resistance

Exosomes as part of the transportation system contain a variety of nuclear acid species, including DNAs, messenger RNAs (mRNAs), microRNAs, long noncoding RNAs (lncRNAs) and circular RNAs (circRNAs). In past decades, there has been an emerging interest in exosomal nucleic acids, however, research on exosomal DNA remains elusive. Exosomal DNA is originated from gDNA in the nucleus and mtDNA in the mitochondria (in culture supernatant of myoblasts) 19, 20. Exosomal DNA is derived from gDNA in the nucleus and mtDNA in the mitochondria (from the culture supernatant of myoblasts). The gDNA of exosomes contains DNA fragments from multiple chromosomes and represents the whole genome of the cell from which the exosome originates, which also includes mutated DNA fragments 19, 21. It has been reported that DNA packaged into exosomes was significantly higher in cancer-derived exosomes compared to exosomes from normal cells and the distribution varies among cancer cell lines 22-24. However, the mechanism of how nuclear DNA or mitochondrial DNA is packaged into exosomes remains unclear. Several studies have reported that excess cytoplasmic DNA in mammalian cells can be secreted via exosomes to inhibit the process of senescence or apoptosis 22, 25. Alternatively, recent researchers have indicated the packaging of gDNA into exosomes could be selective. For example, Domenis et al. reported that exosomes derived from SW480 (human colorectal adenocarcinoma cell line) cells contributed to transferring dsDNA fragments containing the entire coding sequence of both tumor protein p53 (*TP53*) and KRAS proto-oncogene (*KRAS*) genes, harboring the *TP53* c.818G > A and* KRAS* c.35G > T typical mutations. In addition, the stimulation of lipopolysaccharides (LPS) could promote the packaging of the *TP53* gene rather than *KRAS* gene indicating the selective package of gDNA 26. Furthermore, recent studies have also suggested the essential role of exosomal DNA in the development of drug resistance in malignant cells. Sansone et al. found that cancer-associated fibroblasts derived exosomes were loaded with high amounts of mtDNA, resulting in that the mtDNA-acquired recipient cancer stem cells exited metabolic quiescence and transformed into hormone therapy-resistant cancer cells 27.

### 3.2. Exosomal mRNA cargoes and tumor drug resistance

Messenger RNA (mRNA) has been identified as an important part of exosome-originated cargoes mediating various biological functions. Studies have revealed the selective disposal of RNA species into the exosome and the intercellular transfer of mRNA to recipient cells by tumor-derived exosomes 28. Several mechanisms have been implicated in the selective packaging of RNA into the exosomes, including specific RNA sequence motifs or secondary conformations 29-31, different affinities for membrane lipids depending on lipid-bilayer binding motifs within the specific RNA sequences 32-34, and interaction with RNA binding proteins (RBPs), such as heterogeneous nuclear ribonucleoproteins A2/B1 (HNRNPA2B1) 35 and vacuolar protein sorting-associated protein 4A (Vps4A) 36. The transportation of mRNAs to other types of tumor cells was based on clathrin-mediated endocytosis. Furthermore, Skog J et al. and Tannous et al. have confirmed that exosome-originated mRNAs can be translated into functional proteins in recipient cells, and may mediate the cellular transfer of phenotypes 37, 38. Lobb et al. found that non-small cell lung cancer (NSCLC) cell-derived exosomes mediated the transfer of chemoresistance from tumor cells to donor epithelial cells. These pieces of research supported that mRNA can be selectively sorted into exosomes with additional translation, leading to the transfer of drug resistance phenotype besides, studies have identified that the existence of exosomal androgen-receptor splice variant 7 messenger RNA (AR-V7) in Pca was associated with resistance to hormonal therapy 39.

### 3.3. Exosomal noncoding RNA (ncRNA) cargoes and tumor drug resistance

Among the various exosomal nuclear acid cargoes, noncoding RNA (ncRNA) was believed to play a unique role in exosome-induced drug resistance. To date, it has reported that non-coding RNAs loaded in exosomes include miRNA, lncRNA, circRNA, piwi-interacting RNAs (piRNAs), and tRNA-derived small noncoding RNA (tsRNA) 40. The selective packaging of noncoding RNAs by exosomes rather than a simple copy of the cytoplasmic content, coupled with the highly stable structure of exosomes that protects the internal non-coding RNA from degradation, allows the exosomal noncoding RNAs to perform a critical biological function in tumor drug resistance 7, 41, 42.

#### 3.3.1. Exosomal lncRNA cargoes and tumor drug resistance

LncRNAs are a group of RNAs with a length of more than 200 bp that lack open reading frames. They are widely distributed in organisms, participating in the regulation of various physiological and pathological processes. Recently, a growing number of studies have demonstrated the involvement of exosome-derived lncRNAs in the regulation of tumor drug resistance. Unlike lncRNAs in the cytoplasm and the nucleus, exosomes protect exosomal lncRNAs from degradation, allowing them to reach the recipient cell and participate in the regulation of drug resistance. LncRNA-mediated regulation can be carried out at epigenetic level, transcriptional level and post-transcriptional level.

At the epigenetic level, lncRNA-mediated regulation mainly occurs through interactions with epigenetic modifiers that alter the epigenome directly through DNA methylation, post-translational modifications or the structural alterations of chromatin 43. It should be noted that the polycomb repressive complexes (PRCs), a well-studied nucleoprotein complex, promotes gene silencing by altering chromatin structure. It has been reported that about 20% of lncRNAs interact with PRCs. By interacting with different epigenetic factors, especially by silencing tumor suppressor genes, lncRNA have an important role in cancer progression 44.

The regulation of lncRNAs at the transcriptional level often occurs through interactions with transcription factors. LncRNAs can serve as enhancers so that transcription factors are recruited to the promoter regions of genes, which in turn modify the expression of the corresponding genes and enable the regulatory process. For example, linc01134 was found to be one of the most upregulated lncRNAs in oxaliplatin-resistant (OXA-R) hepatocellular cancer cells. The increased linc01134 expression suggested a poor efficacy of OXA. Mechanistically, lnc01134 recruits transcription factor SP1 to the *p62* promoter region and activates the antioxidant pathway. It has been reported that a series of pathways are involved in the linc01134/SP1/*p62* axis mediated-regulation of OXA resistance both *in vitro* and *in vivo*, including altering cell viability, apoptosis, and mitochondrial homeostasis 45.

Moreover, lncRNAs contribute to the regulation of cellular functions at the post-transcriptional level. Depending on the targets of lncRNAs, post-transcriptional regulation can be divided into two main categories: regulation by lncRNA-protein binding and regulation by lncRNA-RNA interactions. In the first category, due to the diverse functions of the proteins bound to lncRNAs, the regulatory effects are complex and extensive. For instance, exosomal lncRNA‐SNHG7 was identified to promote docetaxel resistance of lung adenocarcinoma (LUAD) cells by inducing autophagy in LUAD cells and promoting M2 polarization in macrophages. Mechanistically, it was found that ELAV-like protein 1(ELVA1, also known as HUR), an important RNA binding protein that regulates RNA stability, was recruited by exosomal SNHG7 to stabilize autophagy related gene 5(*ATG5*) and *ATG12* for the induction of autophagy in LUAD cells. In addition, exosomal SNHG7 promotes phosphatase and tensin homolog (PTEN) ubiquitination and activates the phosphoinositide 3-kinase (PI3K)/AKT pathway to induce M2 polarization in macrophages by recruiting cullin 4A (CUL4A), a ubiquitination-associated protein 46. For the second class of regulation, the interaction between lncRNAs and miRNAs is by far the most studied one. MiRNAs are mainly recognized to negatively regulate gene expression by binding to their mRNA targets. LncRNAs can serve as molecular sponges that sequester miRNAs, which in turn reverse the negative regulatory effects by inhibiting miRNA binding to their target mRNAs. Qu et al. found that lncARSR was highly expressed in sunitinib-resistant RCC cells, which was correlated with clinically poor sunitinib response 47. Mechanistically, lncARSR was identified to competitively bind miR-34/miR-449 to prevent *c-MET* and *AXL* from protein (argonaute-2) Ago2-based miRNA-induced expression repression in RCC cells 18. According to current studies targeting exosome-mediated drug resistance, lncRNAs are mainly involved in post-transcriptional regulation that occurs in the cytosol, rather than in transcriptional regulation or epigenetic regulation that takes place in the nucleus, which can be associated with the uptake of exosome by recipient cells through endocytosis 6.

#### 3.3.2. Exosomal circRNA cargoes and tumor drug resistance

Circular RNAs (circRNAs) are a class of non-coding RNAs formed by a back-shearing event in which the downstream 5′ site binds to the upstream 3′ site 48. Due to the widespread presence of circRNAs in exosomes, some studies have suggested that exosomes could be an important pathway for cells to clear endogenous circRNAs 47, 49. However, a growing number of recent studies have highlighted the potential importance of circRNAs, also demonstrating their biogenesis and function. CircRNAs can be characterized by high abundance, conservation stability and prevalence. Due to the absence of 5′ and 3′ ends, circRNAs are inherently resistant to the main enzymes of degradation, which contributes to their higher stability than linear RNAs. Consequently, circRNAs may accumulate in cells and lead to the development of drug resistance and the progression of tumor.

Notably, exosomal circRNA usually functions as competitive endogenous RNA (ceRNA) to regulate drug resistance. The circRNA X-linked inhibitor of apoptosis (circ-XIAP) is derived from the mRNA back-splicing of the *XIAP* gene, which serves as an oncogene in prostate cancer (PCa). Zhang et al. found that circXIAP was upregulated in docetaxel (DTX)-resistant prostate tissue specimens and cell lines. Meanwhile, circXIAP was found to be widely distributed in exosomes secreted by DTX-resistant cells and could be transported via exosomes to DTX-sensitive cells resulting in the acquirement of docetaxel resistance. The underlying mechanism revealed that circXIAP served as a molecular sponge for miR-1182 to promote tumor protein D52 (TPD52) expression. Exosomal circXIAP enhanced DTX-resistant cell proliferation, migration and invasion by regulating the miR-1182/TPD52 axis 50. Moreover, exosomal circUSP7 was reported to induce CD8 + T cell dysfunction and anti-PD1 resistance by regulating the miR-934/ SH2 containing protein tyrosine phosphatase-2 (SHP2) axis in non-small cell lung cancer (NSCLC) 51.

Similar to the post-transcriptional regulation of lncRNAs, circRNAs can also impact tumor drug resistance by interacting with RNA-binding proteins. Xu et al. found that circRNA-SORE was upregulated in sorafenib-resistant hepatocellular carcinoma (HCC) cells and played an important role in sorafenib resistance. CircRNA-SORE was further confirmed to be transported by exosomes to spread sorafenib resistance among HCC cells, and silencing circRNA-SORE could substantially overcome sorafenib resistance. Mechanistically, circRNA-SORE binds to the Y-Box Binding Protein 1(YBX1) in the cytoplasm and prevents the nuclear interaction of YBX1 with the E3 ubiquitin ligase PRP19, thereby blocking the PRP19-mediated degradation of YBX1 52.

Emerging research is now exploiting liquid biopsy techniques to discover novel, highly accurate biomarkers from human body fluids by minimally invasive or non-invasive means 53, 54. Since circRNAs are enriched and stable in exosomes, and exosomal circRNAs usually remain stable in body fluids to label tumors 55, this suggests the potential of exosomal circRNAs as biomarkers for early and minimally invasive cancer diagnosis and prognostic assessment.

#### 3.3.3. Exosomal microRNA cargoes and tumor drug resistance

MicroRNAs (miRNAs) are non-coding, single-stranded RNAs with ~22nt length, which are important post-transcriptional regulators of gene expression. These small RNA molecules regulate gene expression primarily by serving as guide molecules for RNA silencing. Through base-pairing with complementary sequences within the 3′-untranslated region (3′-UTR) of the target mRNA, miRNAs typically downregulate gene expression, mainly in the form of translation inhibition or degradation induction. The corresponding process is accomplished by the RNA-induced silencing complex (RISC) 56, 57. Through direct interactions with mRNAs, miRNAs regulate the transcription and translation of genes within the cell and are thus involved in a wide range of physiological and pathological processes, such as tumorigenesis or tumor drug resistance. Correspondingly, when miRNAs are selectively sorted into exosomes, these miRNAs can be transported to other cells for intercellular regulation. Recent studies have confirmed the presence of several key components of the miRNA processing pathway as well as primary, precursor and mature miRNAs in exosomes, suggesting that exosomes may act as miniature miRNA processing factories, producing mature miRNAs internally 58. In addition, accumulating evidence suggests that, due to their bilayer membrane and nanoscale size, exosomes can protect miRNAs from complement fixation, macrophage clearance or damage, thereby extending their circulating half-life and enhancing their biological activity. In particular, exosome membranes are rich in sphingolipids, ceramides and cholesterol, which contribute to distinguishing exosomes from cell membranes and facilitating their uptake by recipient cells. Overall, exosomes act as carriers and potential processing factories for miRNAs, enabling the specific and persistent regulation of miRNAs between cells 59, 60.

An increasing number of studies have identified that exosomal miRNAs play an extremely broad and far-reaching regulatory role in drug resistance at multiple levels, including facilitating drug efflux, regulating drug target expression, activating signaling pathways related to drug resistance, and so on. For example, in prostate cancer, cancer-associated fibroblasts (CAFs) serve as an essential part of the tumor microenvironment. Shan et al. found that CAF-derived exosomes loaded with miR-423-5p increased PC resistance to taxane. Otherwise, the inhibition of exosomal miR-423-5p enhanced the drug sensitivity of PC cells *in vivo*. Mechanistically, CAF-secreted exosomal miR-423-5p targets gremlin 2 (GREM2) through the activation of transforming growth factor beta (TGF-β) pathway, facilitating the chemoresistance of PC 61. In addition, studies have revealed that low miR-128-3p expression correlates with response to oxaliplatin treatment in advanced colorectal cancer (CRC) patients. The overexpression of miR-128-3p was found to inhibit epithelial-mesenchymal transition (EMT) and increase intracellular accumulation of oxaliplatin in established oxaliplatin-resistant CRC cells. The underlying mechanisms suggest that miR-128-3p could inhibit the oxaliplatin-induced EMT process by silencing *BMI1* (BMI1 Proto-Oncogene, Polycomb Ring Finger), and enhance drug efficacy by targeting multidrug resistance protein 5 (MRP5), a drug transport protein, to reduce the oxaliplatin efflux 62.

### 3.4. Exosomal protein cargoes and tumor drug resistance

Exosomes have an enriched protein content, including a great variety of membrane proteins and soluble proteins in the exosome lumen 63. Different from the simple copy of intracellular proteins, the exosomal package of protein is selective. The protein sorting pathway within the exosome can be divided into ESCRT-dependent and ESCRT-independent pathways 34, 64. The ESCRT-dependent mechanism that sorts proteins into exosomes is involved with ubiquitination while the ESCRT-independent mechanisms include post-translational modifications and lipid-related sorting 64, 65. A large number of these proteins have been confirmed to be involved in the development of drug resistance, varying in their roles in drug resistance according to their localization.

The membrane proteins of exosomes play a crucial role in promoting tumor drug resistance. Exosomal membrane proteins can serve as receptors or ligands to activate drug resistance-associated signaling pathways. Studies have revealed that monoclonal antibodies (mAb) against programmed death-ligand 1 (PD-L1) exhibit outstanding effectiveness in immune checkpoint blockade treatments towards different types of cancer, such as advanced renal cell carcinoma, breast cancer and CRC 66-68. Notably, studies have shown that exosomal PD-L1 can directly bind to anti-PD-L1-mAb contributing to the immunotherapy resistance of tumor cells 69. P-glycoprotein 1 (P-gp) also known as multidrug resistance protein 1 (MDR-1), is an ATP-dependent efflux pump located on the membrane, which can pump exogenous substances such as chemotherapeutic drugs out of cells. Corcoran et al. have reported that variants of docetaxel-resistant prostate cancer cells (DU145RD and 22Rv1RD) are rich in P-gp protein expression, as are the exosomes they secrete. Furthermore, exosomes derived from docetaxel-resistant variants were identified to confer drug resistance to sensitive variants, which may be in part due to exosomal MDR-1/P-gp transfer 70, 71.

**Fig 2:**
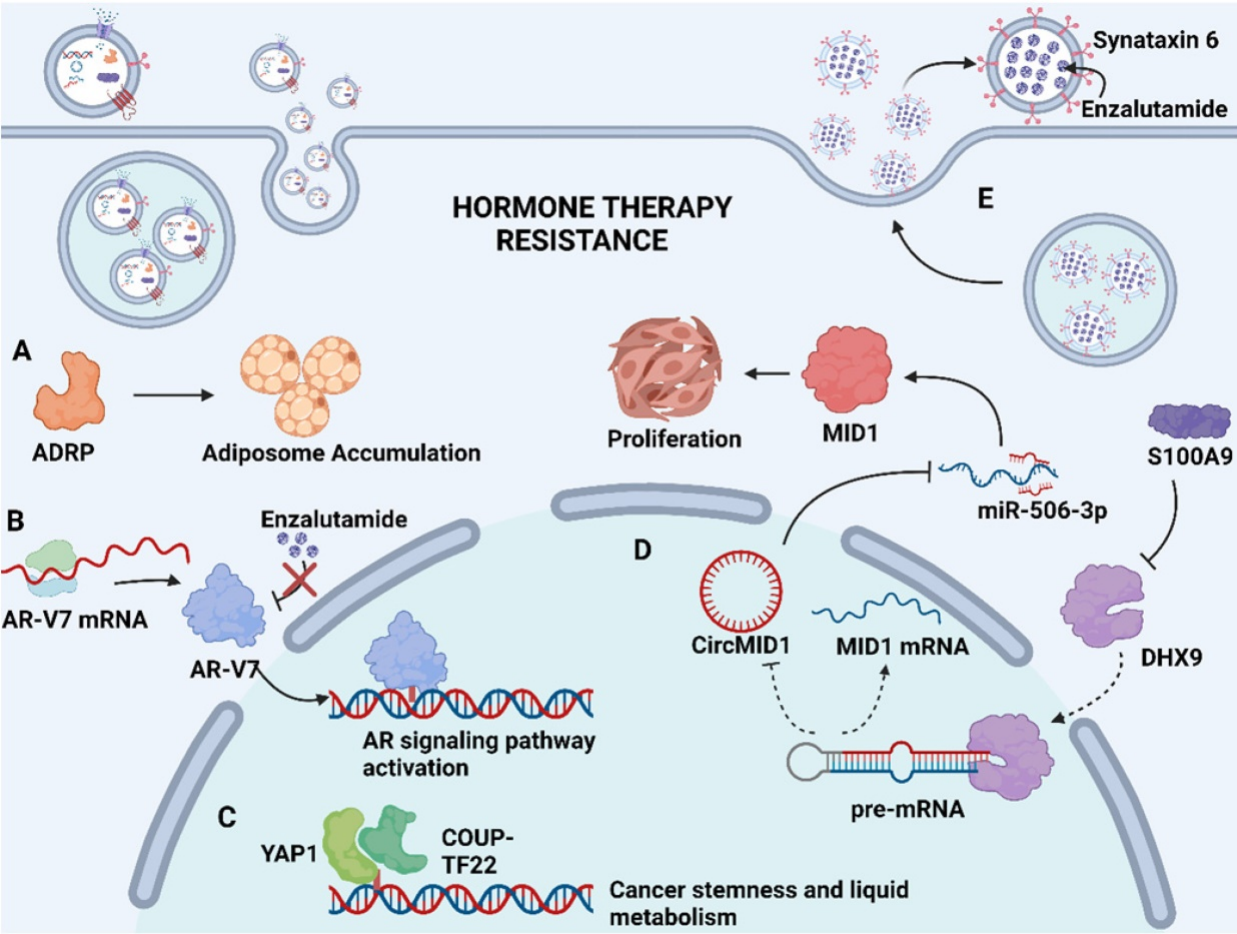
** Mechanism of exosome-mediated resistance to hormone therapy in Pca. (A)** Adipocyte differentiation-related protein (ADRP, a major component of adiposome) was able to cause adiposome accumulation, thereby inducing the neuroendocrine differentiation of Pca. **(B)** Spliced variants 7 of androgen receptor (AR-V7) encode proteins that lack the enzalutamide binding domain but retain the transactivation structural domain, conferring androgen-independent AR transactivation and leading to resistance to enzalutamide. **(C)** Transcriptional factors YAP1 and COUP-TFII coregulate the relative genes expression of cancer stemness and lipid metabolism in prostate cancer, leading to enzalutamide resistance. **(D)** S100A9 (a proinflammatory protein) downregulated the expression of RNA helicase DHX9, thereby promoting the generation of circMID1. CircMID1 functions as a molecular sponge to sequester miR-506-3p, inducing the overexpression of MID1 and contributing to cancer cell proliferation along with hormone therapy resistance. **(E)** Upregulation of syntaxin 6 in enzalutamide-resistant Pca increases the secretion of exosome, thereby mediating the extracellular transfer of enzalutamide and leading to drug resistance. Created with BioRender.com.

Soluble proteins in the exosome lumen also play a significant part in mediating drug resistance. These soluble proteins are selectively packaged into exosomes and transported to the recipient cells to exert their unique physiological effects, such as catalyzing physiological reactions as enzymes, affecting gene expression as transcription factors, or activating downstream signaling pathways as upstream proteins, thereby inducing drug resistance 72, 73. In advanced Pca, neuroendocrine differentiation (NED) is considered to be associated with increased resistance to chemotherapy. Recent findings indicated that adipocyte differentiation-related protein (ADRP, a major component of adiposome) was detected in exosomes derived from castration-resistant prostate cancer (CRPC) cells C4-2 and DU145 that underwent NED (Fig. 2A). The paracrine effect of exosomal ADRP was able to induce NED in adjacent CRPC cells by regulating peroxisome proliferator-activated receptor γ (PPARγ)-elicited adiposome accumulation 74.

### 3.5. Other exosomal cargoes and tumor drug resistance

In addition to proteins and nucleic acids, the exosomal cargo also contains molecular metabolites. These molecular metabolites can be transferred to surrounding cancer cells via exosome and influence the metabolism of recipient cells, promoting cancer progression 75-77. For example, tumor-derived exosomes could transfer prostaglandin E2 (PGE2) and TGF-β to induce the accumulation of myeloid-derived suppressor cells (MDSCs) expressing cyclooxygenase-2 (COX-2), interleukin 6 (IL-6), vascular endothelial growth factor (VEGF) and arginase-1. These expanded myeloid cells contribute to tumor development by providing a supportive stroma and immune evasion 78. Alternative studies have confirmed the existence of glutamate and lactate in exosomes originated from human mesenchymal stem cells (hMSCs). Glutamate can provide precursors for major macromolecular classes through carbon and nitrogen trafficking, while lactate can enhance the survival of cancer cells under hypoxic and nutrient-deficient conditions. In addition, the low pH caused by the secretion of lactate through modified glucose/glutamine metabolism is suggested to be one of the causes of cancer evasion of immune surveillance 79.

Apart from above exosomal cargos, the drug itself or its active metabolites can also be involved in exosome cargos. Through exosome-mediated direct drug efflux, the accumulation of drug in the tumor cell can be insufficient, diminishing drug efficacy and thus inducing drug resistance. Studies have identified that enzalutamide (Enz)-resistant Pca cells secrete more exosome than sensitive Pca cells. The increased secretion of exosome leads to the enhanced extracellular efflux of Enz. Underlying mechanisms revealed that syntaxin 6, a tail-anchored membrane protein involved in membrane fusion events, was significantly upregulated in enzalutamide (Enz)-resistant Pca cells (Fig. 2E). The upregulation of syntaxin 6 strongly increased the secretion of exosome, thereby mediating the extracellular transfer of Enz 17.

## 4. Exosomes in genitourinary cancers

### 4.1. Exosomes and drug resistance in renal cell carcinoma

Kidney cancer is one of the 10 most common types of cancer in both male and female individuals, accounting for 4.1% of all new cancer cases and predominantly affecting males. It is estimated that 79,000 people in the United States will be diagnosed with kidney cancer in 2022 and 13,920 will die from it 1, 80. Renal cell carcinoma (RCC) is the most common form of kidney cancer, accounting for up to 85% of cases. Treatment strategies for RCC include partial or radical nephrectomy, ablation (radiofrequency, cryo- or microwave ablation), and active surveillance 81, 82. While surgical resection can be curative for patients with early-stage RCC, an additional systemic treatment is required for many patients who present with recurrent or potentially metastatic disease upon diagnosis. Considering the limited response rate of RCC to conventional chemotherapy and radiotherapy 83, 84, novel therapies have emerged to treat mRCC, including targeted therapies and new immunotherapeutic agents 80, 85. Despite their improved outcomes, the gradual development of resistance in advanced RCC-affected patients is still a major obstacle limiting the patients' prognosis, in which exosomes also play an important role.

#### 4.1.1. Target therapy resistance in RCC

Among all heterogeneous subtypes of RCC, clear cell RCC (ccRCC) is the most common subtype accounting for 70% to 75% of cases, which correlates closely with alterations in the von Hippel-Lindau (*VHL*) gene. In tumor cells, *VHL* inactivation leads to enhanced hypoxia-inducible factor (HIF) activation, which ultimately results in the overexpression of downstream vascular endothelial growth factor (VEGF) and platelet-derived growth factor (PDGF) 85, 86. The activation of HIF can also be regulated by the mammalian target of rapamycin (mTOR) pathway. For the above-mentioned gene targets, targeted therapies against mTOR and receptor tyrosine kinase (RTK) signaling have emerged in the clinic. Sunitinib, as the first-line treatment for ccRCC, is an oral multi-target RTK inhibitor with satisfying anti-angiogenic effects and direct anti-tumor activity owing to its inhibition of vascular endothelial growth factor receptor (VEGFR), platelet-derived growth factor receptor (PDGFR), stem cell growth factor receptor, and FMS-like tyrosine kinase 3 80, 85. However, most patients with advanced RCC eventually develop acquired resistance to sunitinib therapy, resulting in a significant barrier to improved prognosis for patients with sunitinib. Researchers have found that lncARSR is markedly upregulated in sunitinib-resistant cell lines and the high expression level is associated with poor sunitinib response in RCC patients. LncARSR may function as a ceRNA to sequester miR-34 and miR-449, which leads to the liberation of AXL and c-MET, contributing to the sunitinib resistance of RCC cell lines. lncARSR could be transferred by exosome to recipient cells, thereby preventing the miRNA-mediated inhibition of AXL and c-MET expression. Additionally, in sunitinib-resistant cells, exosomal lncARSR can also promote the expression of AXL and c-MET, which leads to sunitinib resistance in RCC cells 18. In addition to the involvement in sunitinib resistance, exosomes also participate in the initiation of other targeted drug resistance processes. Xuan et al. reported that the low expression of miR-549a in sorafenib-resistant cells and exosomes contributes to the upregulation of HIF1α in endothelial cells, inducing vascular permeability and angiogenesis to promote tumor metastasis 87. Tsuruda et al. showed that exosomes derived from renal carcinoma cells facilitate resistance in tumors cells against mTOR inhibitor rapamycin via the mTOR/ERK (extracellular signal-regulated kinase)/STAT (signal transducer and activator of transcription)/NF-κB (Nuclear factor-κB) signaling pathway 88.

#### 4.1.2. Immunotherapy resistance in RCC

In terms of immunotherapy, interleukin-2 and interferon alpha (IFNα) were some of the first agents for the systemic treatment of RCC, but with only moderate success 89. Emerging immunotherapeutic strategies have been explored for the treatment of patients with advanced or metastatic RCC. Recently discovered immune checkpoint-based therapeutic agents, such as programmed cell death protein 1 (PD-1)/programmed death ligand 1 (PD-L1) and cytotoxic T lymphocyte-associated antigen 4 (CTLA-4) checkpoint inhibitors, have become an integral part of the therapeutic management of advanced or mRCC 90, 91. Nevertheless, immunotherapeutic resistance may develop through the tumor derived exosome-mediated modulation of lymphoid cell function. Studies have confirmed that exosomes derived from human kidney adenocarcinoma ACHN cells could inhibit the human immortalized line of Jurkat T lymphocytes proliferation *in vitro* by triggering apoptosis and inhibiting cytokine production in Jurkat T lymphocytes. The underlying mechanisms suggested that tumor-derived exosomes contain soluble Fas ligands. By binding to Fas receptors, these ligands activate the caspase pathway, contributing to the induction of apoptosis 92. In addition to lymphoid cells, regulating the function of myeloid cells (myeloid-derived suppressor cells (MDSCs), tumor-associated macrophages (TAMs)) also contributes to the immunotherapy evasion in RCC. MDSCs are a heterogeneous group of immature myeloid cells (IMCs) with strong immunosuppressive patterns and functions 93. Gao et al. found that renal cancer-derived exosomes could induce the expansion and activation of MDSC in a toll-like receptor2 (TLR2)-dependent manner. Moreover, exosome-activated MDSCs could exert an antigen-specific immunosuppressive effect on cytotoxic T lymphocytes (CTLs), contributing to the immunotherapeutic resistance of renal cancer 94. Similar to MDSC, M2-polarized macrophages could also exert immunosuppressive functions and promote tumor growth. Scholars have demonstrated that exosomal circSAFB2 promotes M2 macrophage polarization through the miR-620/ Janus kinase 1 (JAK1)/STAT3 axis, leading to the immune evasion of RCC 95.

### 4.2. Exosomes and drug resistance in prostate carcinoma

Prostate cancer is the most common cancer and the leading cause of cancer-related deaths in men worldwide. It is estimated that there will be 268,490 new cases diagnosed and 34,500 deaths caused by Pca in 2022 in the USA 1. When diagnosed with local Pca, patients will be recommended to receive therapies such as radical prostatectomy or radiotherapy. However, some patients with local advanced or metastatic Pca will receive androgen deprivation therapy (ADT) through surgical orchiectomy (castration) or medical castration (using either a gonadotropin-releasing hormone (GnRH) agonist or a GnRH antagonist) to block androgen function, since androgen is a crucial factor for Pca development. Although ADT therapy is initially effective for advanced Pca, the vast majority of patients eventually show progress while receiving ADT, and the disease state is referred to as castration-resistant prostate cancer (CRPC). Chemotherapy such as docetaxel and second-generation antiandrogen drugs, including enzalutamide, abiraterone and apalutamide, has shown the improvement of survival benefit for CRPC patients, resulting in approved therapeutic options worldwide 96, 97. Unfortunately, cancer recurrence still occurs and eventually results in poor prognosis. Exosomes have been reported to participate in the development of resistance to chemotherapy and hormone therapy in Pca, which may pave the way for their use as potential therapeutic targets to overcome resistance in Pca.

#### 4.2.1. Hormone therapy resistance in Pca

Hormone therapy is a mainstream treatment approach for Pca due to the crucial role of androgens in Pca development. The transformation from hormone-sensitive prostate carcinoma (HSPC) to CRPC is the critical milestone of Pca progression, which exhibits distinct genomic and proteomic landscapes. The exosome-mediated transfer of proteins and nuclear acids may serve an important role in hormone resistance (Fig. 2). It has been demonstrated that exosomal S100A9 (a proinflammatory protein) derived from MDSCs facilitates castration-resistant prostate cancer progression via circMID1/miR-506-3p/MID1 pathways 98 (Fig. 2D). Alternative studies reported that the exosomal miR-146a-5p expression level was significantly downregulated under ADT. The loss of miR-146a-5p may enhance epithelial-mesenchymal transition (EMT), thereby accelerating the metastasis of cancer cells by regulating the epidermal growth factor receptor (EGFR)/ERK pathway 99.

Hormone therapy resistance may also occur when patients are treated with antiandrogen drugs. Enzalutamide and abiraterone are both inhibitors of the androgen signaling pathway, blocking the AR signaling pathway by competitively binding to androgen receptors and depleting intratumoral and adrenal androgens, respectively. Although enzalutamide and abiraterone are exploited as alternative treatment options for CRPC patients, almost all patients inevitably acquire drug resistance. A reasonable explanation regarding resistance to both drugs may involve the presence of androgen receptor splice variants. Alternatively, spliced variants encode proteins that lack the ligand-binding structural domain but retain the transactivation structural domain, such that these generated truncated proteins lack the enzalutamide binding site and confer androgen-independent AR transactivation, leading to resistance to both agents 100, 101 (Fig. 2B). Recent studies have identified the existence of AR and its splice variants AR-V7 and AR-V4 in exosomes derived from Pca, which was associated with resistance to novel hormonal agents 16, 39. Researchers have also investigated alternative pathways for exosomal cargoes contributing to the development of drug resistance. Lee, H.-C., et al. found that exosomal yes1 associated transcriptional regulator (YAP1) and chicken ovalbumin upstream promoter transcription factor II (COUP-TFII) facilitated the development of enzalutamide resistance through the induction of cancer stemness and lipid metabolism in prostate cancer 102 (Fig. 2C). In addition, Peak, T. C., et al. showed that the upregulation of syntaxin 6 increased the secretion of exosomes, leading to the extracellular transfer of enzalutamide 17 (Fig. 2E).

#### 4.2.2. Chemotherapy resistance in Pca

Taxanes are the only chemotherapy agents that significantly prolong overall survival in clinical trials in patients with metastatic CRPC, which makes docetaxel as the first-line therapy for CRPC treatment. Nevertheless, the vast majority of patients administered with docetaxel eventually acquire chemoresistance, accompanied by a high mortality rate. The involvement of exosomes in the development of chemoresistance can be divided into two major patterns. Firstly, exosomes can promote the efflux of drugs from tumor cells. Recent studies have reported that P-gp, an adenosine triphosphate (ATP) -dependent efflux pump located on the membrane, can be detected at higher levels in the extracellular vesicles of docetaxel-resistant Pca cells and are potentially higher in the serum exosomes of patients who have acquired docetaxel resistance 70, 103. In addition to the exosome-mediated transfer of P-gp, exosomes also transfer functional cargoes that activate anti-apoptosis signaling, leading to resistance to chemotherapy. It has been found that miR-27a is significantly upregulated under treatment with cisplatin, doxorubicin and docetaxel in Pca tissues. Further studies have confirmed the existence of miR-27a in exosomes derived from primary prostate fibroblasts (PSC27 cells). Exploring the relevant mechanisms revealed that exosomal miR-27a may facilitate chemoresistance in Pca by restraining the expression of *P53* gene and exerting a pro-survival function 104.

### 4.3. Exosomes and drug resistance in urothelial carcinoma of the bladder

UC is the seventh most common type of cancer in population. According to statistics, there will be approximately 81,180 new cases and 17,100 deaths from UC in 2022 in the U.S. 1. Based on the depth of tumor tissue invasion, UC can be classified as non-muscle invasive bladder cancer (NMIBC) and muscle invasive bladder cancer (MIBC). The main treatment for NMIBC is transurethral resection of the bladder tumor plus intravesical chemotherapy. In contrast, patients with MIBC are generally treated with radical cystectomy and neoadjuvant chemotherapy. The novel combination of cisplatin adjuvant chemotherapy showed an improved overall survival of patients. Combination chemotherapy based on cisplatin is currently considered as a first-line treatment for advanced and metastatic UC. However, chemoresistance is a major obstacle to improved patient prognosis.

Similar to prostate cancer, exosomes are involved in the development of chemotherapy resistance in UC. They may transfer biologically active cargo to regulate the expression of P-gp protein, thereby promoting drug efflux to generate chemoresistance. Researchers have revealed that exosomal lnc00355 derived from cancer-associated fibroblasts (CAFs) facilitates cisplatin resistance in bladder cancer. Lnc00355 can serve as a molecular sponge to sequester miR-34b-5p, thereby upregulating the expression of downstream P-gp protein. Thus, exosomal lnc00355 promotes BC cell resistance to cisplatin through the lnc00355/miR-34b-5p/ATP binding cassette subfamily B member 1(ABCB1) axis 105. On the other hand, exosomes may activate specific signaling pathways to induce chemoresistance. A previous study indicated the presence of miRNA-148b-3p in exosomes derived from CAFs, and the increased expression of miRNA-148b-3p promoted the metastasis and chemoresistance of bladder cancer cells. Mechanistically, PTEN was confirmed as a downstream target of miR-148b-3p. Exosomal miRNA-148b-3p could inhibit the expression of PTEN, thereby activating the Wnt/β-catenin pathway, resulting in chemoresistance and metastasis 106. In addition to the above pathway, studies have reported that exosomes promote the chemoresistance of bladder cancer cells by enhancing their EMT 41, 106.

## 5. Strategy based on the inhibition of exosome secretion

Given the deep involvement of exosomes in the drug resistance process, the strategy based on the inhibition of exosome formation and release to overcome drug resistance has emerged 107. Since the first report of neutral sphingomyelinase inhibitor GW4869 can inhibit the release of exosomes 108, a large number of novel agents towards exosome secretion inhibition has been discovered, such as γ-Tocotrienol 109, bisindolylmaleimide‐I 110 and chloramidine 108. It is found that exosome secretion inhibitors could not only reverse the exosome induced drug resistance but also show the cooperative therapeutic efficacy in overcoming the immunotherapy resistance combined with PD-L1 antibody 111, 112. Recently, Im et al. identify that sulfisoxazole, an FDA-approved oral antibiotic shows significant anti-tumor effect through inhibition of small extracellular vesicle secretion 113. The gradually expanding class of exosome inhibiting agents holds great promise in overcoming drug resistance.

## 6. Exosomes as biomarkers in liquid biopsies

There is a growing interest in the application of liquid biopsies to detect biomarkers in urologic malignancies. Liquid biopsy encompasses minimally invasive or non-invasive tests performed on biofluid samples, including blood or urine, to detect molecules derived from cancer 114. Owing to their minimally invasive nature, liquid biopsies allow for serial sampling, resulting in their potential application in detecting minimal residual disease (MRD) or recurrence, tracking tumor progression and predicting drug resistance or therapeutic response in solid tumors and hematologic malignancies.

Recently, exosomes have emerged as novel analytes in liquid biopsies, demonstrating unique advantages as biomarkers. Firstly, they provide a rich and complete set of circulating RNA biomarkers. In contrast to circulating tumor DNA, exosomes as analytes can supply multi-component information including tumor-associated proteins, circulating RNA as well as DNA. The ability of exosomes to protect their cargoes from degradation and the enrichment of tumor-specific molecules allow exosomes to provide more detailed information 115-117. Besides giving valuable information on gene expression levels and tumor-specific somatic alterations, exosomal RNA also provides additional opportunities to investigate other processes that may indicate cancer progression or therapeutic response 117. For instance, numerous studies have reported that the detection of exosomal AR-V7 mRNA in Pca is associated with resistance to hormonal therapy 39, 118, 119. Other studies have also reported that CD44v8-10 mRNA contained in serum exosomes can serve as a diagnostic marker for docetaxel resistance 120. The second advantage of exosomes lies in their ability to enrich tumor-associated signals. As with other liquid biopsy methods, distinguishing tumor signals from normal cellular “noise” remains a challenge when analyzing liquid biopsy samples, especially when extracting proteins and nuclear acids directly from urine, as secreted proteins and nuclear acids from the urinary tract are abundant and can produce an overwhelming background that interferes with the analysis 8, 121. However, the sources of exosomes can be recognized by specific markers on their surface. Exosomes can be enriched from biological fluids using surface markers, and further enrichment of exosome subpopulations from different tissue sources can be achieved using tumor-specific or tumor-enriched surface marker proteins. Thus far, several studies have supported the feasibility of this approach. Urine contains a mixture of EVs from several parts of the genitourinary tract, and studies to date have identified multiple uEV markers characterizing different structures of the urinary tract 122-124. In addition, Hoshino et al. conducted the proteomic profiling of 426 human samples and identified tumor-derived EV markers from human tissues and plasma that differed from normal controls, which might be useful for tumor detection and exosome isolation 125.

Large-scale liquid biopsies of urinary exosome biomarkers can provide important insights into the therapeutic monitoring of genitourinary cancers. However, developing exosome-based minimally invasive liquid biopsies faces considerable challenges. In this regard, the major task lies in the selection of standard isolation and analysis methods of exosomes from urine. Several methods have been developed by researchers for the isolation of urinary exosomes, including ultracentrifugation, ultrafiltration and size exclusion chromatography. Obviously, there are differences in the purity and yield of exosomes obtained by different isolation methods, and usually one aspect improves at the expense of the other. Moreover, the selection of different isolation methods has an impact on the characteristics and analytical results of both exosomes and contaminants. Therefore, the diversity of disease scenarios and the dynamic molecular composition of clinical samples may require a variety of options for the isolation and analysis of urinary exosomes; a single standardized method for urine collection, urinary exosome isolation, and analysis is unlikely to effectively cover all disease scenarios and problems. The absence of standard methods has particularly complicated the development of reference standards for urine exosome analysis. To design, optimize and upgrade protocols for practical clinical applications, researchers are required to understand the impact of different preanalytical variables on the nature and quality of isolated urinary exosomes prior to sample analysis. In order to move the field of urinary exosome research forward, the establishment of reference standards for urinary exosomes is essential, which requires ongoing collaborative discussions between industry, regulatory agencies and standards bodies.

## 7. Conclusion

Despite significant advances in modern antineoplastic drugs, the development of drug resistance often leads to failure in the treatment of genitourinary malignancies. In this review, we summarize recent investigations on the role of exosomes in drug resistance in genitourinary cancers. Data shows that the profiling of exosomal cargoes differs after the acquirement of drug resistance, and the exosome-mediated transfer of cargoes could greatly contribute to cancer progression and drug resistance. These findings highlight the importance of exosomes and provide a better understanding of drug resistance. Due to the unique biological role in drug resistance, exosomes may be used as candidate biomarkers for predicting and monitoring therapeutic efficacy in patients with genitourinary cancer, thus occupying an important position in the future of tumor detection, prediction, and treatment.

Despite the broad clinical application prospects, several challenges must be resolved before exosome-based diagnosis or prognosis are applied in clinic. The first major limitation lies in the absence of standardized exosome isolation techniques and related quality controls, thus making it difficult to establish an authoritative evaluation system. Meanwhile, the second limitation is the lack of large prospective studies to support the clinical translation of exosome liquid biopsies. Although exosome-based liquid biopsy appears promising, there are still demands for more research before exosome can be incorporated into clinical applications.

## Figures and Tables

**Table 1:** Mechanisms underlying drug resistance mediated by exosomes in genitourinary cancer

Origin of exosome	Cancer type	Exosome cargo	Target(s)	Drug resistance	Mechanism	Reference
sunitinib-resistant RCC cell lines 7Su3rd and ACSu3rd	RCC	lncARSR	AXL and c-MET	sunitinib resistance	sequestering miR-34 and miR-449, leading to the upregulation of AXL/c-MET and the activation of STAT3, AKT, and ERK signaling	18
sorafenib-resistant RCC cell line (786-O-SR)	RCC	low expression of miR-549a	HIF-1α	sorafenib resistance	inducing vascular permeability and angiogenesis to promote tumor metastasis	87
renal cell adenocarcinoma cell lines (769-P)	RCC	unknown	mTOR	mTOR inhibitor rapamycin resistance	activating the mTOR-ERK1/2-STAT-NF-κB signaling	88
kidney adenocarcinoma cells ACHN	RCC	Fas ligands	Fas receptors	immunosuppression	triggering Jurkat T cell apoptosis and contributing to immune evasion	92
mouse renal adenocarcinoma cell line RENCA	RCC	HSP70	TLR2	immunosuppression	activating the MDSC and suppressing the cytotoxic effect of CTL	94
RCC	RCC	circSAFB2	JAK1/STAT3 signaling pathway	immunosuppression	promoting M2 macrophage polarization	95
MDSCs	Pca	S100A9	MID1	hormone therapy resistance	facilitating castration-resistant prostate cancer progression via circMID1/miR-506-3p/MID1 pathways	98
cancer-associated fibroblasts	Pca	loss of miR-146a-5p	EGFR	hormone therapy resistance	promoting the EMT to accelerate cancer cell metastasis by modulating the EGFR/ERK pathway	99
CRPC	Pca	AR-V7, AR-V4	AR signaling pathway	hormone therapy resistance	androgen-independent AR transactivation	16, 39
enzalutamide-resistant prostate cancer cells	Pca	YAP1 and COUP-TFII	genes related to cancer stemness	enzalutamide resistance	promoting enzalutamide resistance through induction of cancer stemness and lipid metabolism	102
enzalutamide-resistant PCa cells (C4-2B, CWR-R1, and LNCaP)	Pca	enzalutamide	enzalutamide	enzalutamide resistance	high expression of syntaxin 6 leading to increased exosomal secretion of enzalutamide	17
enzalutamide-resistant PCa cells (C4-2B MDVR)	Pca	ADRP	PPARγ-ADRP-mediated adiposome accumulation	neuroendocrine differentiation	stimulating adiposome accumulation through PPARγ-ADRP pathway	74
primary prostate fibroblasts (PSC27 cells)	Pca	miR-27a	Trp53	chemoresistance	restraining the expression of P53 gene and inhibiting the apoptosis caused by chemotherapy	104
DTX-resistant prostate cancer cell lines (DU145/DTX and PC3/DTX),	Pca	Circ-XIAP	TPD52	docetaxel resistance	suppressing LKB1/ AMPK-mediated autophagy	50
cancer-associated fibroblasts	Pca	miR-423-5p	GREM2	taxane resistance	activating the TGF-β pathway	61
docetaxel-resistant Pca cells	Pca	P-gp	docetaxel	docetaxel resistance	increasing the drug efflux of docetaxel	70, 103
cancer-associated fibroblasts	UC	lnc00355	P-gp	cisplatin resistance	sequestering the miR-34b-5p and upregulating the expression of P-gp, leading to increase of drug efflux	105
cancer associated fibroblasts	UC	miR-148b-3p	PTEN	chemosensitivity	Inhibiting the expression of PTEN, thereby activating the Wnt/β-catenin pathway, resulting in chemoresistance and metastasis	106

**Abbreviations**: STAT3, signal transducer and activator of transcription 3; ERK, extracellular signal-regulated kinase; HIF-1α, hypoxia-inducible factors; mTOR, mammalian target of rapamycin; HSP70, the 70 kilodalton heat shock proteins; TLR2, toll like receptor2; MDSCs, myeloid-derived suppressor cells; RCC, renal cell carcinoma; JAK1, Janus kinase1; EGFR, epidermal growth factor receptor; CRPC, castration-resistant prostate cancer; AR, androgen receptor; YAP1, Yes-Associated Protein 1; ADRP, adipocyte differentiation-related protein; TRP53, transformation-related protein 53; DTX, docetaxel; TPD52, tumor protein D52; LKB1, liver kinase B1; AMPK, adenosine monophosphate-activated protein kinase; Pca, prostate cancer; UC, urothelial carcinoma of the bladder; PTEN, phosphatase and tensin homolog.

## Abbreviations

RCC: renal cell carcinoma
UC: urothelial carcinoma of the bladder
Pca: prostate carcinoma
TURP: transurethral resection of the prostate
EVs: extracellular vehicles
MVBs: multivesicular bodies
ILVS: intra luminal vesicles
ESCRT: endosomal sorting complex required for transport
ISEV: International Society of Extracellular Vesicles
VHL: von Hippel-Lindau
ccRCC: clear cell renal cell carcinoma
HIF: hypoxia-inducible factor
VEGF: vascular endothelial growth factor
PDGF: platelet-derived growth factor
mTOR: mammalian target of rapamycin
RTK: receptor tyrosine kinase
VEGFR: vascular endothelial growth factor receptor
PDGFR: platelet-derived growth factor receptor
PD-1: programmed cell death protein 1
PD-L1: programmed death ligand 1
CTLA-4: cytotoxic T lymphocyte-associated antigen 4
MDSCs: myeloid-derived suppressor cells
TAMs: tumor-associated macrophages
IMCs: immature myeloid cells
TLR2: toll-likereceptor2
CTLs: cytotoxic T lymphocytes
ADT: androgen deprivation therapy
GnRH: gonadotropin-releasing hormone
CRPC: castration-resistant prostate cancer
HSPC: hormone-sensitive prostate carcinoma
EMT: epithelial-mesenchymal transition
NMIBC: non-muscle invasive bladder cancer
MIBC: muscle invasive bladder cancer
CAFs: cancer-associated fibroblasts
MRD: minimal residual disease
TSG101: tumor susceptibility gene 101
Vps4A: vacuolar protein sorting-associated protein 4A
ATG5: autophagy related gene 5
PI3K: phosphoinositide 3-kinase
CUL4A: cullin 4A
PTEN: phosphatase and tensin homolog
TPD52: tumor protein D52
SHP2: SH2 containing protein tyrosine phosphatase-2
JAK1: Janus kinase 1
EGFR: epidermal growth factor receptor
YAP1: yes1 associated transcriptional regulator
COUP-TFII: chicken ovalbumin upstream promoter transcription factor II
ATP: adenosine triphosphate
YBX1: Y-Box Binding Protein 1
MRP5: multidrug resistance protein 5
TGF-β: transforming growth factor beta
GREM2: gremlin 2
BMI1: BMI1 Proto-Oncogene
PD-L1: programmed death-ligand 1
COX-2: cyclooxygenase-2
IL-6: interleukin 6
VEGF: vascular endothelial growth factor
IFNα: interferon alpha
mTOR: mammalian target of rapamycin
ERK: extracellular signal-regulated kinase
STAT: signal transducer and activator of transcription
NF-κB: Nuclear factor-κB
